# Mechanical and Structural Evaluation of the PA12 Desktop Selective Laser Sintering Printed Parts Regarding Printing Strategy

**DOI:** 10.1089/3dp.2020.0111

**Published:** 2021-08-04

**Authors:** Magdalena Tomanik, Matylda Żmudzińska, Magdalena Wojtków

**Affiliations:** Department of Mechanics, Materials Science and Biomedical Engineering, Wroclaw University of Science and Technology, Wroclaw, Poland.

**Keywords:** printing strategy, polyamide powders, mechanical properties, porosity, fractography, SLS

## Abstract

Despite the dynamic development of additive manufacturing technologies, including selective laser sintering (SLS), there is still limited information on the impact of key factors in printing strategy, on the properties of three-dimensional (3D) printed parts. Such factors, such as the orientation of printed layers toward the powder bed or elements target dimensions, seem to be particularly important, from both a mechanical and a structural point of view. Besides, the scientific articles mainly focus on the analysis of one type of loading condition in the samples, that is, the uniaxial tensile test, which were printed on industrial SLS printers. This is a considerable limitation because very often not only tensile forces but also compressive forces act on the structural elements. Therefore, this study aimed at evaluating the influence of desktop SLS printed parts' orientation and diameter on their structural and mechanical parameters. The mechanical properties of samples printed from PA12 powder on the desktop SLS 3D printer were tested in uniaxial tensile and compression tests, as well as structural properties were investigated. For the purposes of this article, 5 angular orientations of the samples in relation to the powder bed and three diameters of cylindrical samples were analyzed. The research has shown that in the case of samples subjected to tensile load, the printing strategy is important, and the best mechanical parameters are obtained for parts printed at an angle of 0°, that is, in the powder bed's plane. The highest values of mechanical parameters were obtained for a part oriented at an angle of 0°. In the case of the uniaxial compression test and structural parameters, the parts orientation turned out to be an insignificant factor affecting the tested parameters. However, the diameter of printed elements was proven to have a significant influence; the best geometric and dimensional representation was observed for parts biggest in size.

## Introduction

Due to the dynamic development of additive manufacturing (AM) technologies, including selective laser sintering (SLS), they are increasingly used in a wide range of industries, also as a source of constructional elements. Despite the available information on mechanical properties of the elements printed from polyamide powders, the data declared by the material suppliers include only results obtained in the uniaxial tensile test. Besides, an important problem is the lack of information about the part's orientation in the build chamber, and consequently about the dependence of the main load axis on the orientation of the sintered layers in the model.

The polymer powder used in the SLS method is polyamide PA12, which in the specific form, produced by EOS is PA2200. The declared range of values of mechanical parameters of parts made of polyamide used in SLS printers, given in technical specifications of material suppliers, is as follows: Young's modulus (E) equals 1586–1650 MPa, ultimate tensile strength (TS) equals 43–48 MPa, and elongation at break (EaB) equals 14–20%.^1–3^ Provider of desktop SLS printer LISA, Sinterit for PA12 polymer, on the other hand, gives the following properties: *E* = 1029 MPa, TS = 41 MPa, EaB = 13%.^4^ The values were obtained for three-dimensional printed (3DP) samples in each case.

One of the most frequently analyzed cases of printing strategy in the literature focuses on the orientation of the parts during manufacturing and its influence on the mechanical properties of 3DP parts, printed with industrial SLS printers. Configurations investigated most commonly are parallel or perpendicular layers to the long axis of the sample,^5–9^ although there are also analyses considering a greater number of configurations, such as 0°, 45°, 90°,^10^ 0°, 20°, 45°, 90°,^11^ or different.^12,13^ The printing strategy suggested by SLS printers producers indicates that the parts should be oriented at a certain angle to the powder bed. However, this angle is not clearly indicated, especially in the context of obtaining the highest mechanical strength or structural properties of printed elements.

Moreover, Stoia *et al.*^11^ have shown that the arrangement of the models in the build chamber does not affect the properties obtained in the uniaxial tensile test. However, Adam and Zimmer^14^ indicated the significant influence of model arrangement during printing on the obtained dimensional accuracy of elements. According to the results, the smallest dimensional deviations of the part in relation to the Computer Aided Design (CAD) geometric model were reached for the angle of 90°.

One of the major issues within SLS technology is limited information on the influence of the printing process, in particular the thermal processes, on the final structure of the polyamide molecules. Therefore, it is difficult to predict the final mechanical, structural, and geometric parameters of 3DP parts. Dadbakhsh *et al.*^15^ showed no differences in the obtained morphometric parameters of virgin powder, used and mixed virgin and used in the ratio 1:1. However, a significant effect of the type of powder or its mixture on the final structure of printed parts and their mechanical parameters were observed.

The porosity of 3DP parts seems to be one of the decisive factors influencing mechanical parameters.^16^ According to Flodberg *et al.*,^17^ internal defects of the powder structure constitute 43% of the porosity in the part. Those defects may decide on mechanical properties, and influence the fracture mechanism of the sample, including the fracture crack propagation that may be observed in the scanning electron microscope (SEM) microscopy (fractography).^18^

In the case of parts made of polyamide powders, the analyses conducted so far, usually regards uniaxial tensile tests,^5–8^ although there are also reports in which samples were subjected to complex loading conditions^12,17^ or where due to the characteristics of the AM technology, a complex state of stress was taken into account.^19^ There are still little reports in the literature about the testing of SLS printed samples subjected to compressive forces. This aspect is particularly important because of the diverse nature of the load conditions to which structural elements are subjected during operation.

Considering literature analysis, no attempts on the assessment of parts printed on desktop three-dimensional (3D) SLS printers were carried out, despite the differences (e.g., laser source, scanning patterns) between industrial and desktop machines. Nowadays, desktop SLS printers are getting popular because of the compromise between their price and accuracy. Moreover, according to the authors' knowledge, the influence of more than two types of 3DP parts angular orientation during printing on the mechanical characteristics, including both uniaxial tensile and compression loading, has not been tested. Another important factor is the compression properties assessment, which is rarely investigated, especially considering the part size and orientation in the build chamber.

Therefore, the aim of this study was to:

- perform mechanical and structural analysis of parts printed on the desktop SLS printer;
- analyze the significance of printing orientation and size on compression 3D printed samples;
- analyze the significance of printing orientation and define the fracture mechanism of tensile 3D printed samples.

## Materials and Methods

### Materials

The polyamide powder used in the study was a PA12 (Sinterit, Krakow, Poland), with a granulation of 18–90 μm. It was a mixture of virgin powder (completely new) and used PA12 powder in a 30:70 ratio. Two types of samples were printed for mechanical and structural tests: dogbone samples (*n* = 15) according to ISO 527 and cylindrical samples (*n* = 15) with diameters *d* = 3, 6, and 9 mm. The cylindrical samples for the compression test were cut to a height corresponding to 1.5d (according to ISO 604) with a precision cut-off machine (Accutom-5; Struers, Copenhagen, Denmark).

**FIG. 1.**
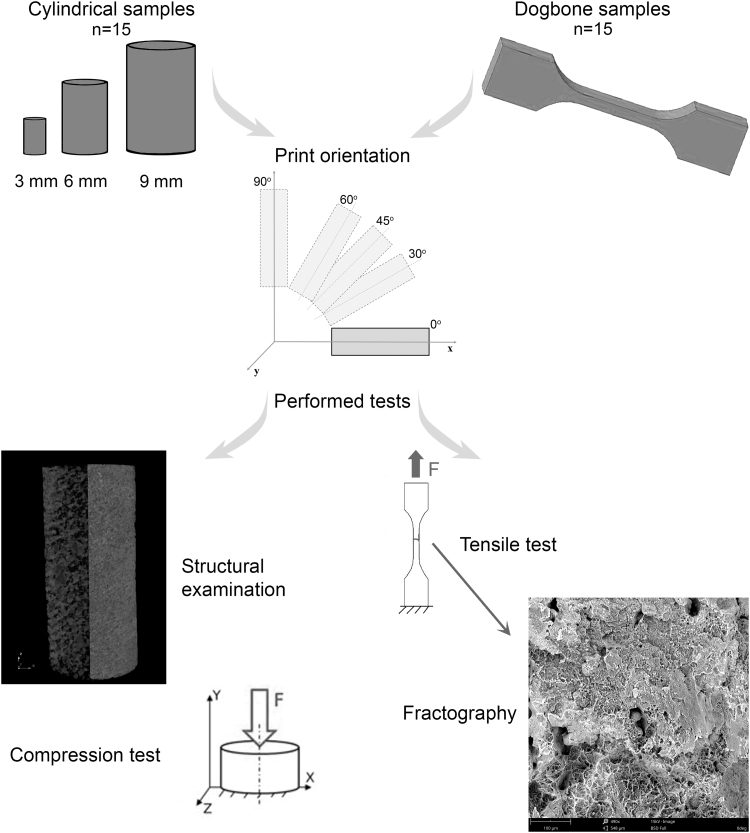
The flow of the experiment, including material and methodology.

All models were printed with a layer thickness of 0.175 mm, in the following angular orientations (α): 0°, 30°, 45°, 60°, 90° in relation to the powder bed plane. The printing process was carried out on Lisa desktop SLS 3D printer (Sinterit) by using an IR laser (λ = 808 nm) with a power of 5 W. After finishing the printing process, the chamber was left to cool down completely, for no less than 12 h. The obtained elements were subjected to postprocessing by using glass balls with a granulation equal to 200 μm. The experimental setup, including sample orientations, is presented in Figure 1.

### Mechanical examination

Mechanical investigations, including uniaxial tensile and static compression tests, were performed on the MTS MiniBionix 858 (MTS Corporation), in accordance with ISO 527 and ISO 604, respectively. Based on the calculated stress–strain (σ = *f(ɛ)*) curves ultimate TS (MPa), compression strength (CS [MPa]), Young's modulus for compression (E_C_ [MPa]), and tension (E_T_ [MPa]) followed by EaB (%) were determined.

### Structural examination

To carry out a structural analysis of printed cylindrical samples (for the compression test), a high-resolution x-ray computer microtomography SkyScan 1172 (Bruker, Kontich, Belgium) was used. The scanning process was conducted with the following X-ray tube parameters: 40 kV, 250 μA, and image pixel size 7.4 μm. After the projection was registered, it was reconstructed by using nRecon software (Bruker). The cross-sections allowed to conduct 3D structural analysis (CTAn; Bruker) and determine the following parameters: total porosity (Po(tot)); eccentricity (Ecc) describing to what extent the cross-section of the examined object deviates from a perfect circle (for which the value of Ecc equals 0); and connectivity within the structure as a fragmentation index (Fr.I).

Dimensional deviations (ΔØ) of the part in relation to the CAD geometric model were determined by measuring the diameter of each cylindrical sample 10 times using a caliper (Mitutoyo, Aurora) and relating the obtained results to the intended diameter.

The fractographic analysis of dogbone samples was performed with the use of the scanning electron microscope SEM (Phenom ProX; Phenom-World, Eindhoven, Netherlands). The microscopic analysis was conducted on the area of destruction.

### Statistical analysis

The statistical analysis was performed by using Prism 7 software (GraphPad Software, San Diego, CA). Grubb's test was used to identify the outlines. The normal distribution of the analyzed parameters has been verified by using the Shapiro-Wilk normality test. The significance of the influence of the 3DP parts' orientation on the obtained parameters was verified by using the Friedman test with Dunn's *post hoc*. Moreover, the influence of the diameter of analyzed samples on mechanical and structural parameters was evaluated by using the One-way Repeated Measurements Anova test with Tukey's *post hoc*. All analyses were performed at the level of significance *p* < 0.05. The results were presented in the form of mean values with standard deviations.

## Results

### Tensile test

**FIG. 2.**
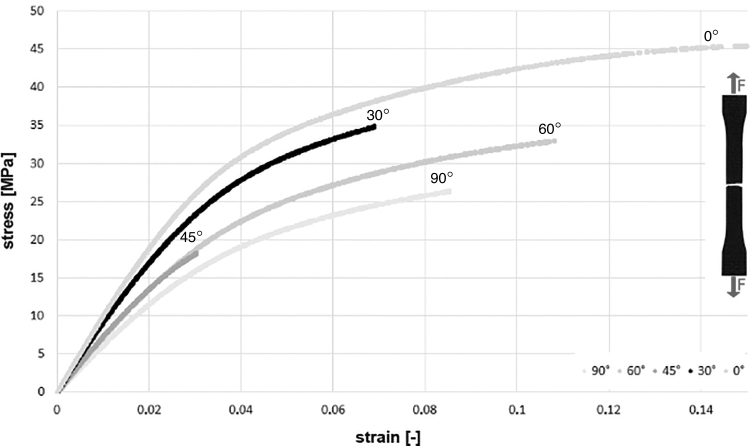
Examples of stress–strain curves for samples with different angular orientations (α) obtained in the uniaxial tensile test.

The stress–strain curves obtained in the uniaxial tensile test for particular angular orientations showed similar characteristics (Fig. 2). The break occurred in the middle part of the measurement section of dogbone samples.

The highest TS value, on average 42.5 ± 3.1 MPa, was obtained for printouts oriented at an angle of 0° to the printer bed (Table 1), whereas values calculated for 45° were more than twice lower (*p* = 0.0451). The ultimate TS of parts printed at an angle of 30° and 60° were similar, and they reached 28.1 and 25.6 MPa, respectively.

**Table 1.** Mechanical Parameters of PA12 Printouts Obtained in the Uniaxial Tensile Test

Angle (°)	Tensile test		
	TS (MPa)	E_T_ (MPa)	EaB (%)
0	42.5 ± 3.1^a^	864 ± 72^b^	13.1 ± 2.3^a^
30	28.1 ± 8.4	690 ± 143	6.7 ± 1.6
45	16.0 ± 2.3	613 ± 27	2.7 ± 0.3
60	25.6 ± 8.9	694 ± 32	8.4 ± 5.7
90	17.1 ± 10.0	426 ± 150	6.0 ± 3.4

Values are presented as means ± SD. Differences between the analyzed printout orientations were examined by using the Friedman test (Dunn *post hoc*).

^a^ Statistical significance between 0° and 45°.

^b^ Statistical significance between 0° and 90°.

EaB, elongation at break; E_T_, tensile Young's modulus; TS, tensile strength.

In the case of E_T_, a statistically significant difference was observed between the orientation 0° and 90° (*p* = 0.0195), where the values were 864 and 426 MPa, respectively. Tensile Young's modulus of parts with angles equal 30° and 60° were characterized by the same dependence as in the case of TS.

Maximal EaB, with an average value of 13%, was again obtained for samples printed at 0°. A significant decrease in this parameter, amounting to as much as 80%, was shown for an angle of 45° (*p* = 0.0195). A similar EaB value of ∼6% was observed for prints at α = 30° and α = 90°.

### Compression test

The analysis showed no influence of 3DP parts orientation on the mechanical properties, therefore the analysis was performed in three groups of different sample diameters (*n* = 5 samples in the group). The summary results of mechanical and structural properties are presented in Table 2.

**Table 2.** Values of Mechanical Parameters and Structural Printouts from PA12 Obtained in the Uniaxial Compression Test

Diameter (mm)	Compression test			Structural characteristic	
	CS (MPa)	E_C_ (MPa)	Po(tot) (%)	Ecc (−)	Fr.I (1/mm)
3	118.3 ± 19.6^a^	692 ± 59^a,b^	11.6 ± 1.5^b^	0.20 ± 0.01^a,b^	−23.80 ± 9.59^a,b^
6	134.8 ± 11.9^c^	1408 ± 60^c^	4.9 ± 0.4^c^	0.15 ± 0.04	−62.15 ± 31.27^c^
9	68.1 ± 3.3	1106 ± 13	33.8 ± 4.7	0.13 ± 0.02	−103.11 ± 7.46

Values are presented as means ± SD. Differences between the analyzed cylindrical printout diameters were examined by using the repeated-measurement one-way analysis of variance test (Tukey *post hoc*).

^a^ Statistical significance between 3 and 9 mm.

^b^ Statistical significance between 3 and 6 mm.

^c^ Statistical significance between 6 and 9 mm.

CS, compression strength; E_C_, compression Young's modulus; Ecc, eccentricity; Fr.I, fragmentation index; Po(tot), total porosity.

Samples with a diameter of 6 mm had the highest CS value of 134.8 MPa, whereas samples with a diameter of 9 mm had the lowest value of 68.1 MPa (*p* = 0.0003). For E_C_, the highest value (1408 MPa) was again obtained for 6 mm samples, and the lowest value (692 MPa) was obtained for 3 mm samples (*p* < 0.0001).

**FIG. 3.**
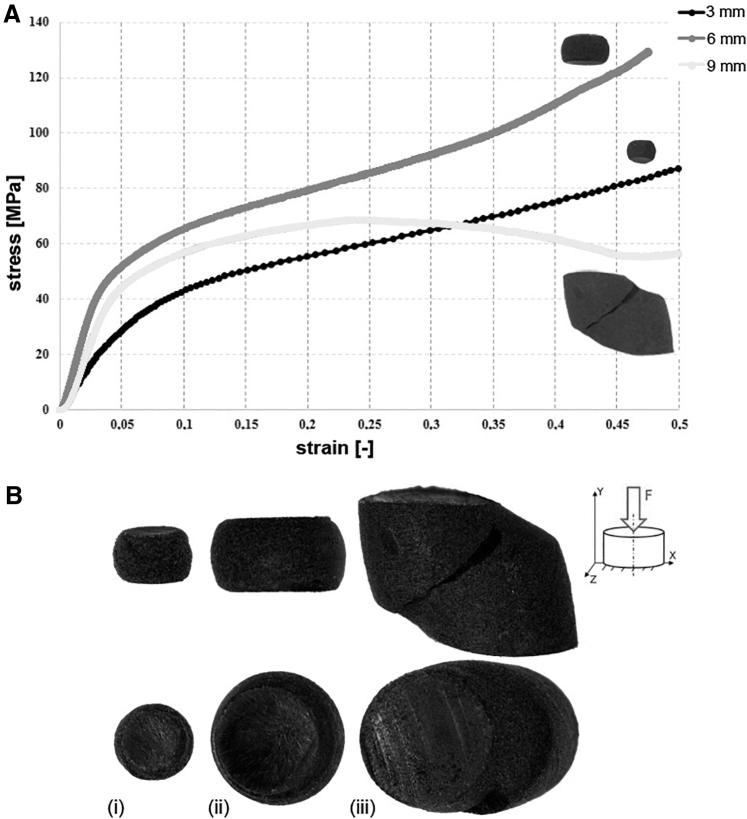
**(A)** Exemplary stress–strain curves obtained in the uniaxial compression test showing a different nature of load bearing by samples of different diameter. **(B)** Samples with diameters of 3 mm **(i)**, 6 mm **(ii)**, and 9 mm **(iii)** presented in xy (*top row*) and xz (*bottom row*) planes. It is important to note the different characteristics of the cylindrical samples fracture between 3 and 6 mm diameters (barrel-shaped) and 9 mm diameter (angular shear, representing slippage at the grain boundary).

However, by analyzing the stress–strain curves, the difference in load-bearing behavior between groups can be observed (Fig. 3A). Curves obtained for 3 and 6 mm samples were alike, whereas the curve for the sample with a diameter of 9 mm had a different shape. Moreover, the same trend was observed in the form of the destruction of individual cylinders (Fig. 3B). Samples with a diameter of 3 and 6 mm take on a barrel-shaped geometry at the end of the test, whereas those with a diameter of 9 mm have a different shape, and characteristic diagonal cracks (Fig. 3B-iii) are observed.

### Structural characteristic

**FIG. 4.**
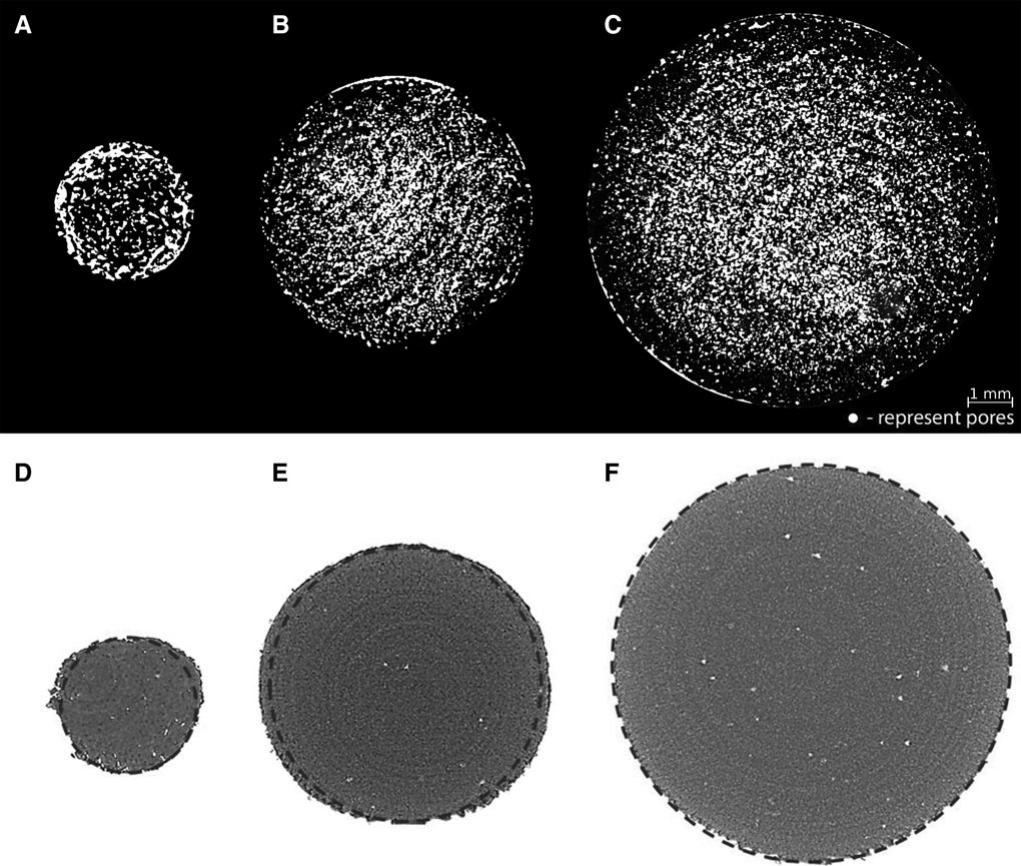
**(A–C)** Visualization of the porosity degree in 3DP parts with differentiation of the diameter of the elements; **(D–F)** cross-sections of 3DP cylinders with marked *dashed* contours corresponding to the designed diameter of the CAD model. 3DP, three-dimensional printed; CAD, Computer-Aided Design.

In the evaluation of the structural parameters of printouts, the key factor was to determine the degree of porosity of the printed parts (Fig. 4A–C). The lowest porosity equal to 5% was characteristic for the 3DP elements with a diameter of 6 mm. The porosity of the 3 mm samples was 12% (*p* = 0.0077) and 34% for 9 mm (*p* < 0.0001 in relation to other sizes). The value of the connectivity parameter Fr.I decreases as the sample diameter increases.

The highest dimensional deviation (ΔØ) was obtained for 3 mm samples. The highest ΔØ differentiation regarding the sample orientation was also observed for this diameter. Elements printed out in orientations 45° and 90° reached the highest deviation from the CAD model, whereas the lowest was noted for parts printed at the 30° orientation angle. Moreover, the highest Ecc values obtained for parts with a diameter of 3 mm indicate that their diameter becomes more elliptical than round, especially in the case of samples printed at an angle of 0° (Fig. 4D–F).

For 6 mm samples, the lowest percentage of the dimensional deviation occurs for the 90° orientation, and the highest for the 30° angle, unlike the 3 mm diameter sample. Elements with a diameter of 9 mm had the best geometric representation (the lowest Ecc values) and the lowest dimensional deviations that did not exceed 1.25% (Table 3).

**Table 3.** Geometrical and Dimensional Representation of Samples with Different Diameters Printed in Different Strategies

Angle (°)	Diameter (mm)					
	3		6		9	
	***Δ***Ø (%)	Ecc (−)	***Δ***Ø (%)	Ecc (−)	***Δ***Ø (%)	Ecc (−)
0	−2.74 ± 1.83	0.38	3.85 ± 0.86	0.17	1.25 ± 0.54	0.14
30	−0.72 ± 0.06	0.19	6.51 ± 0.77	0.20	1.20 ± 0.53	0.14
45	−8.88 ± 1.04	0.20	2.77 ± 0.57	0.17	0.35 ± 0.12	0.14
60	−6.67 ± 0.89	0.20	2.13 ± 0.69	0.12	0.03 ± 0.01	0.11
90	−8.69 ± 1.92	0.22	−1.01 ± 0.58	0.12	0.34 ± 0.11	0.14

Negative percentages in the table mean that the sample dimensions were larger than nominal.

**Δ**Ø, dimensional deviation; Ecc, eccentricity.

**FIG. 5.**
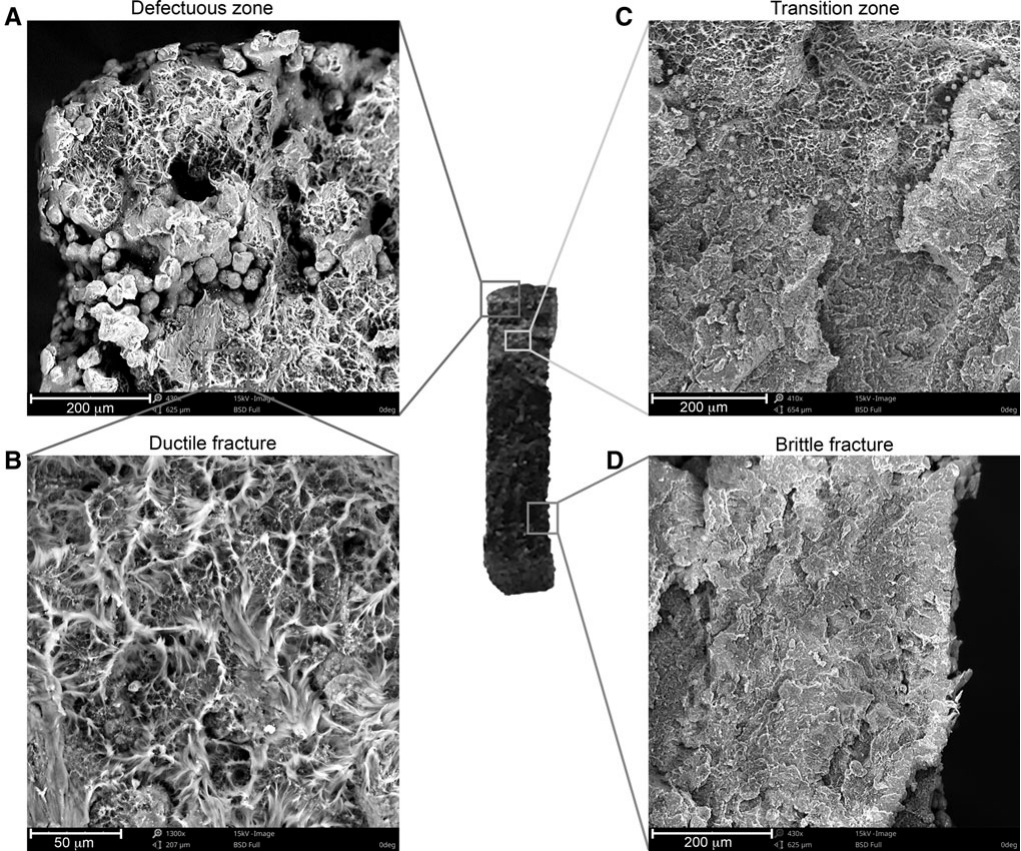
SEM photographs representing different fracture mechanisms in the cross-section of the destruction area of dogbone samples. **(A)** Defectuous zone containing unsintered particles of powder and its magnification **(B)** focusing on the ductile fracture zone. **(C)** Transition zone presenting the border between the ductile and brittle zone; **(D)** Example of the brittle fracture zone. SEM, scanning electron microscope.

As shown in Figure 5, the fractographic analysis of the area of destruction in dogbone samples revealed different fracture mechanisms in its individual regions. Numerous groups of nonmelted powder particles (Fig. 5A) and the ductile fracture (Fig. 5A, B) were observed in the area of the shorter edge of the samples cross-section. Closer to the cross-section center, a *transition zone* with the visible boundary between plastic failure and brittle failure was observed (Fig. 5C, the yellow dotted line indicates the border). By moving the field of view along the long edge of the sample cross-section, an image characteristic of brittle fracture was observed (Fig. 5D). The aforementioned area of ductile fracture occurred only on one side of the sample cross-section. The observations were independent of the build orientation and similar for all of the samples.

## Discussion

The conducted research allowed to evaluate the influence of the SLS printed elements arrangement within the build chamber and its size on the mechanical and structural properties. The authors of this article decided to carry out mechanical tests simulating the most common load conditions of the elements; thus, uniaxial tensile tests as well as compression tests were carried out. The fractographic analysis allowed to identify the types of failure occurring in the samples during the tensile load. Moreover, the use of computer microtomography allowed to evaluate the porosity along with the geometric and dimensional accuracy of the printed parts.

The highest mechanical properties were observed for samples oriented at 0° to the powder bed, in the uniaxial tensile test. Obtained ultimate TS and EaB are in agreement with the parameters declared by the powder supplier (Sinterit),^4^ however the measured Young's modulus is about 15% lower. None of the other samples reached the declared mechanical properties, with the lowest tensile Young's modulus value reaching only 40% of the expected value, obtained for a sample printed at a 90° angle. This may suggest that the mechanical parameters given by the manufacturers apply to samples printed at a 0° angle. Similar relations between the values were described by Hofland *et al.*,^8^ Caulfield *et al.*,^5^ and Wegner and Witt.^6^ In the mentioned papers, only two print orientations (0° and 90°) were analyzed and a noticeable decrease in mechanical parameters (E_T_, EaB, TS) was observed in the samples oriented at a 90° angle in comparison to the 0° samples.

In the study conducted by Stoia *et al.*,^11^ where a larger number of parts orientations were investigated, the described tendency is also visible, however with a decrease of a few percentage points. In such an orientation (90°), a laser is sintering the particles perpendicular to the loading direction, which results in small interference between layers. Failure in those samples occurs due to inter-layer pores. As Faes *et al.*^13^ claim, cracks are initiated at the side of samples and propagate toward the middle. Thanks to the small inter-layer connectivity area, failure in samples printed at 90° occurs at the lowest reached stress and strain levels, in comparison to other directions.

Similarly, lower values of mechanical parameters were obtained for samples printed at 30° and 45° angles. However, the shapes of σ = *f(ɛ)* curves of particular samples indicate the same load-bearing mechanism, regardless of the orientation in the build chamber. Therefore, it can be concluded that in the case of SLS printed elements undergoing tensile loading, the build orientation in the build chamber has little importance, in terms of the mechanical parameters values.

In the case of compression loading, no differences in mechanical parameters were observed depending on the orientation of the part, in contrast to the sample dimensions, where significant variability was observed. Young's modulus values for 6 and 9 mm samples are comparable to those declared by the powder supplier, whereas Young's modulus values for 3 mm samples are ∼50% lower. A different relationship has been observed for CS. Samples with diameters of 3 and 6 mm had a similar ultimate CS value, whereas a value lower by about 50% was recorded for 9 mm samples. In this case (ø = 9 mm), the shape of the stress–strain curves (Fig. 3A) and the fracture mechanisms of the samples (Fig. 3B) were also different. In contrast to the others, they had some characteristics of the curves of the brittle material. Justifications can be found in the internal structure of the tested 3DP elements.

Samples with a diameter of 9 mm were three times more porous than those with a diameter of 3 mm, and six times higher than 6 mm samples. Moreover, connectivity within the structure, described by Fr.I parameter, shows that the greater the diameter, the lower the obtained value; hence, the structure within the sample is less homogeneous. The porosity obtained in the 9 mm samples (Po(tot) = 33.8% ± 4.7%) may result in the introduction of a shear stress component at the material's grain boundaries under compression. Slippage occurring at grain boundaries results in a different fracture mechanism than in the samples with smaller diameters and lower porosity. This may explain the different load-bearing behavior and is confirmed by the characteristic geometry of the sample after destruction. However, this phenomenon requires further research.

The influence of porosity on the load-bearing mechanism was also observed in the case of the CS. The higher the porosity of the sample, the lower the CS.

Nonetheless, the porosity and CS values are likely to be affected by thermal phenomena that occur in the samples during the printing process. When 3DP are printed with the same scan speed, better internal adherence of the material parts is obtained when longer interaction time between particles and laser beam is achieved. A longer time of exposure of one layer results in higher energy transferred to the materials.^20^ Therefore, elements with larger geometrical dimensions (e.g., 9 mm) have a better dimensional deviation and eccentricity than, for example, elements with a diameter of 3 mm. The poorest geometric representation was obtained for the smallest elements printed at an angle of 0°, where the highest value of eccentricity was measured.

It has been observed that the larger the printed object, the less sensitive its geometry is to the printing strategy. Moreover, to avoid heat concentration and therefore warpage of the printed parts, manufacturers suggest printing elements at some angle, but they also implement specific scanning patterns.^21^

It is worth noticing that the porosity obtained for 9 mm samples is significantly higher than for other sizes. In the authors' opinion, such conditions are connected with the accumulation of internal structural defects related to improperly sintered powder, not present to such an extent in smaller samples. This observation is consistent with Flodberg *et al.*,^17^ who indicate that such flaws may constitute more than 40% of the obtained porosity of the prints. Moreover, the relatively high porosity obtained in this research is most likely due to the used printer. A desktop printer consisting of a diode laser was applied here, not an industrial printer with a CO_2_ or other high-power laser. Due to the fact that, to the knowledge of the authors, no studies on desktop printers have been published so far, it is difficult to directly relate the porosity obtained by us to the works of other authors.

The fractographic analysis showed heterogeneity of the dogbone samples in the area of failure, which may indicate structural heterogeneity in the whole sample volume. Areas, where sintering has proceeded adequately, were characterized by brittle failure, and therefore good adhesion between the layers. The poorly sintered areas (partially/nonmelted) contained visible powder particles and were characterized by ductile failure.

The observed differences are also visible on the macro scale, where a different color of the destruction area is observed, and the so-called *transition zone* may also be found. This brighter region can be associated with defects of sintered powder structure, which can result in weaker adhesion between the layers, and also be the place where the fracture begins. Analogous phenomena were described by Crespo *et al.*,^18^ who also described the simultaneous occurrence of two types of damage occurring in PA12 sintered samples, depending on obtained sintering. Likewise, they observed the brittle fracture in properly sintered areas and ductile fracture in inadequately remelted areas.

The presented results refer to the properties of the 3DP parts obtained on the Sinterit Lisa SLS desktop printer. Due to differences in applied technological solutions of particular devices working in SLS technology, the measured structural and mechanical properties can have no counterparts in parts printed on different systems, especially industrial devices. Moreover, an important factor influencing the quality of printed elements is the moisture content of powder, which was not analyzed in this article.^22,23^ Further, according to Gonzalez-Henriques,^20^ differences in the laser source and coloring of the powder might also affect the heat input and crystallization behavior, which might need further investigation.

## Conclusion

The conducted research showed that in the case of SLS printed parts, subjected to tensile forces, an important issue affecting their mechanical strength is the arrangement of models in a build chamber during the printing process. The best mechanical properties were obtained for samples oriented at an angle of 0°, that is, parallel to the printer bed plane. However, the elements subjected to compression loading did not show any influence of the orientation of the elements on the mechanical parameters' values. The size of the printed samples proved to be significant. Six millimeter diameter samples have shown the best mechanical properties and the smallest porosity. The highest geometrical and dimensional accuracy was observed for the biggest elements.

Conducted research proved that in the comparison between industrial and desktop SLS printers there are no significant differences in parts' mechanical properties. However, in terms of structural analysis, visible differences may be found.
